# Fabrication of Ceramic Microchannels with Periodic Corrugated Microstructures as Catalyst Support for Hydrogen Production via Diamond Wire Sawing

**DOI:** 10.3390/ma17112535

**Published:** 2024-05-24

**Authors:** Xinying Li, Chao Gao, Ding Yuan, Yuanbao Qin, Dongbi Fu, Xiyang Jiang, Wei Zhou

**Affiliations:** Pen-Tung Sah Institute of Micro-Nano Science and Technology, Xiamen University, Xiamen 361005, China; 19920190154066@stu.xmu.edu.cn (X.L.); 19920221151574@stu.xmu.edu.cn (C.G.); stu_yuand@163.com (D.Y.); 34520212201660@stu.xmu.edu.cn (Y.Q.); amspl2580123@126.com (D.F.); junglebae14@163.com (X.J.)

**Keywords:** diamond wire sawing, periodic corrugated microstructure, ceramic microchannel, catalyst support, hydrogen production

## Abstract

Hydrogen energy is the clean energy with the most potential in the 21st century. The microchannel reactor for methanol steam reforming (MSR) is one of the effective ways to obtain hydrogen. Ceramic materials have the advantages of high temperature resistance, corrosion resistance, and high mechanical strength, and are ideal materials for preparing the catalyst support in microchannel reactors. However, the structure of ceramic materials is hard and brittle, and the feature size of microchannel is generally not more than 1 mm, which is difficult to process using traditional processing methods. Diamond wire saw processing technology is mainly used in the slicing of hard and brittle materials such as sapphire and silicon. In this paper, a microchannel with a periodic corrugated microstructure was fabricated on a ceramic plate using diamond wire sawing, and then as a catalyst support when used in a microreactor for MSR hydrogen production. The effects of wire speed and feed speed on the amplitude and period size of the periodic corrugated microstructure were studied using a single-factor experiment. The microchannel surface morphology was observed via SEM and a 3D confocal laser microscope under different processing parameters. The microchannel samples obtained under different processing parameters were supported by a multiple impregnation method. The loading strength of the catalyst was tested via a strong wind purge experiment. The experimental results show that the periodic corrugated microstructure can significantly enhance the load strength of the catalyst. The microchannel catalyst support with the periodic corrugated microstructure was put into the microreactor for a hydrogen production experiment, and a good hydrogen production effect was obtained. The experimental results have a positive guiding effect on promoting ceramic materials as the microchannel catalyst support for the development of hydrogen energy.

## 1. Introduction

Hydrogen is one of the most promising clean energy sources in the 21st century with the advantages of being highly efficient and environmentally friendly. However, hydrogen as a secondary energy source is not easy to obtain and store [1]. Hydrogen production via methanol steam reforming (MSR) is an effective means to obtain hydrogen [2]. Methanol has a wide range of sources, a mild reaction temperature (200~300 °C), and a high hydrogen content [3,4,5]. In addition, hydrogen production via MSR can be achieved on site, avoiding expensive and complicated hydrogen storage and transportation [6]. The microchannel reactor is characterized by a small volume, a large specific surface area, and a strong ability to transfer mass and heat. In recent years, the use of a microreactor for MSR as a hydrogen generator to produce hydrogen and to provide a hydrogen source for hydrogen energy vehicles, heavy trucks, ships and aircrafts, and other hydrogen devices and scenarios is a research hotspot [7,8,9,10].

Ceramic materials have better temperature resistance, mechanical strength, and corrosion resistance, making them an ideal material to use as catalyst support in MSR microreactors for hydrogen production [11,12,13]. However, the brittleness and good corrosion resistance make it difficult to process ceramic materials via either traditional machining or chemical etching, and the micro size structure of the microchannels further increases the difficulty of manufacturing, limiting the application of ceramic materials in microchannel reactors to a certain extent [14,15]. In addition, the catalyst support needs to have a good catalyst adhesion ability, which generally requires the surface of the catalyst support to have some rough secondary microstructure so that the catalyst can be firmly loaded onto it, which furthers the manufacturing difficulty of the ceramic material.

At present, researchers have developed a number of ceramic material surface microchannel-processing methods, including injection molding [16,17,18], laser micro-machining [19,20], electric discharge machining [21], micro-milling processing [22,23], and so on. Among them, injection molding technology is favored for its simple process and high forming precision. By precisely controlling the raw material ratio and sintering temperature, the structural integrity and performance stability of the microchannel can be ensured. Due to the limitations of the process of mold manufacturing and demolding, this method faces certain challenges in the processing of high-aspect-ratio microchannels [24,25]. Laser micromachining technology is known for its non-contact processing and wide applicability. The fabrication of complex shape microchannels can be realized via micro-machining ceramic materials with high energy laser beams [26,27,28]. However, the tilt of the heat-affected zone and microchannel sidewall that may occur during laser processing still needs to be further solved [29]. EDM technology is suitable for the processing of various hard materials, including ceramics [30]. It removes material through the phenomenon of electrical discharge between electrodes, thus enabling the precise fabrication of microchannels. The problems of a low material removal rate and a long processing time limit its application in large-scale productions. In addition, micro-milling technology also plays an important role in ceramic microchannel machining [31,32,33]. By coating the surface of the milling cutter with diamond abrasive, the technology can achieve the efficient cutting of ceramic materials. For microchannel machining with high precision and complex shapes, further exploration and optimization of the machining parameters and process flow are still needed. Although the above methods can process the ceramic microchannel support, they are generally complicated and have a low efficiency and high cost. In particular, the need to process ceramic microchannels with secondary microstructures remains a daunting challenge.

Diamond wire sawing (DWS) technology is a commonly used technique in the production of cutting brittle materials such as KDP, monocrystalline/polycrystalline silicon, sapphire, and so on [34,35,36]. This technique involves depositing fine diamond abrasives on the surface of stainless steel wire, utilizing the reciprocating motion of the wire to create repeated scratches on the workpiece’s surface, thereby achieving material cutting. Not only is this technology easy to operate with a high cutting efficiency, but it also ensures superior product quality while reducing environmental pollution [37,38,39,40,41]. As a result, it has gained great popularity. In the process of reciprocating motion, the surface of the material processed via diamond wire sawing may form reciprocating grains, although this is usually not desired in the processing of wafer slices. However, for the ceramic microchannel support, this may provide a very good microstructure, which can improve the loading performance of the catalyst. The comparison between DWS and other ceramic microchannel fabrication techniques can be seen in Table 1.

Based on previous research, the ceramic microchannel catalyst support with a periodic corrugated microstructure was fabricated through diamond wire sawing. The surface morphology of the samples with different processing parameters was observed and characterized with SEM and 3D laser confocal microscopy. The catalyst loading strength of the ceramic support was tested. The relationship between the catalyst load strength and the periodic corrugated microstructure was established. The relevant evaluation indexes are put forward. Finally, the hydrogen production performance and stability of the ceramic microchannel catalyst support with the periodic corrugated microstructure were evaluated. The experimental results provide a new method for promoting the manufacturing technology of ceramic-based catalyst support and its application in the field of new energy.

## 2. Experiments

### 2.1. Materials and Methods

**Figure 1:**
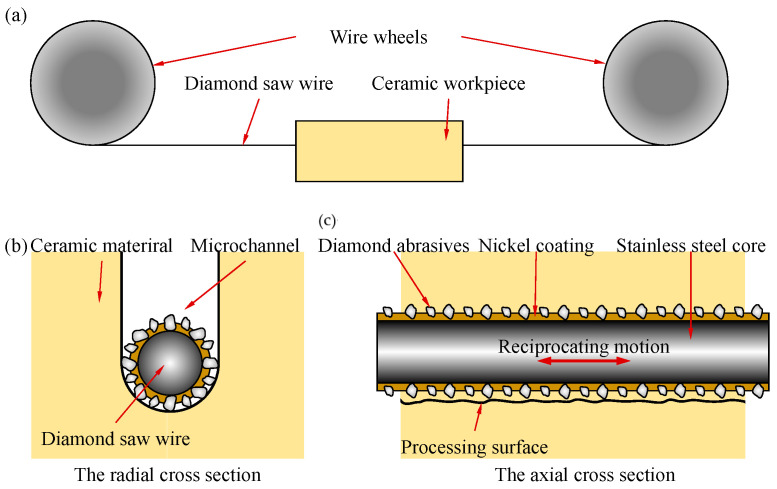
(**a**) The working principle of diamond wire sawing; (**b**) The radial cross section of diamond wire sawing; (**c**) The axial cross section of diamond wire sawing.

**Figure 2:**
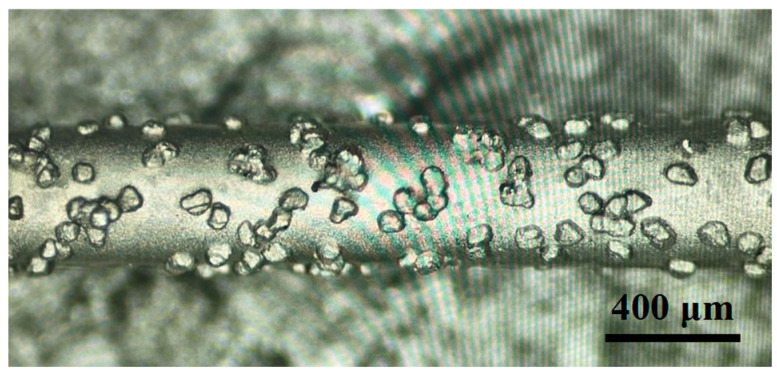
Diamond saw wire.

The ceramic workpiece used in this paper is alumina ceramic; the purity is 99.99%, and the thickness of the workpiece is 2 mm. The material parameters of alumina ceramic are shown in Table 2. Figure 1 shows the working principle of diamond wire sawing. Throughout this process, the saw wire is driven by the line wheel to form reciprocating movements. The saw wire is plated with a layer of diamond abrasive particles. In the wire movement process, the abrasive particles continuously scratch the surface of the workpiece, making the hard and brittle material undergo plastic shear deformation or brittle collapse, so that the material is then removed. In this experiment, a saw wire with a diameter of 450 μm was used for processing, as shown in Figure 2. The density of the diamond abrasive particles on the saw wire was about 30 grits/mm^2^, the average diameter of the abrasive particles was 25 μm, and the size range was 10 μm.

The spindle speed of the diamond wire saw machine used in this experiment has 7 levels, and the feed speed has 5 levels in total. Among them, the spindle speed level is set by the factory and cannot be changed, and the feed speed can be set according to the experimental needs, as shown in Table 3. A single-factor experiment was adopted to study the influence of the processing parameters on the surface topography of the microchannel, and the parameters used in the experiment are shown in Table 4. After processing, the surface morphology of the ceramic microchannel was observed with scanning electron microscopy (JSM-IT500A, JEOL, Tokyo, Japan). The amplitude and ware length of the periodic corrugated microstructure was measured using 3D laser confocal microscope (VK-X1000, Keyence, Osaka, Japan).

### 2.2. Catalyst Loading Performance Test

Multiple impregnation is used to load the catalyst onto the surface of the ceramic support. The preparation process of the catalyst precursor solution refers to previous research works of our research group [6,21,27]. The support surface is cleaned with alcohol to remove impurities before loading. The support is completely immersed in the catalyst solution, and then taken out and dried in the oven for 20 min, resulting in three loadings. Finally, the catalyst-loaded support is baked in a muffle furnace for a more solid catalyst coating.

Before coating the catalyst, the mass of the ceramic support was weighed. The weight of the ceramic support was weighed again after the catalyst was loaded and calcined. The difference between the two is the actual loading mass of the catalyst. In this experiment, ultrasonic vibration was used to simulate the external interference and then to test the strength of the catalyst coating. The ultrasonic cleaners (JP-020, Skymen, Shenzhen, China) were chosen to generate ultrasonic vibration. The ultrasonic cleaning machine was filled with the right amount of water, then a beaker with two-thirds of the volume of deionized water was put into the ultrasonic cleaners, and then the ceramic support loaded with the catalyst and calcined was put into the beaker. The ultrasonic power and frequency were 120 W and 40 kHz, respectively. The vibration time was 90 s. After drying, the weight of the support was weighed and compared with that before the vibration.

### 2.3. Hydrogen Production Performance Test

**Figure 3:**
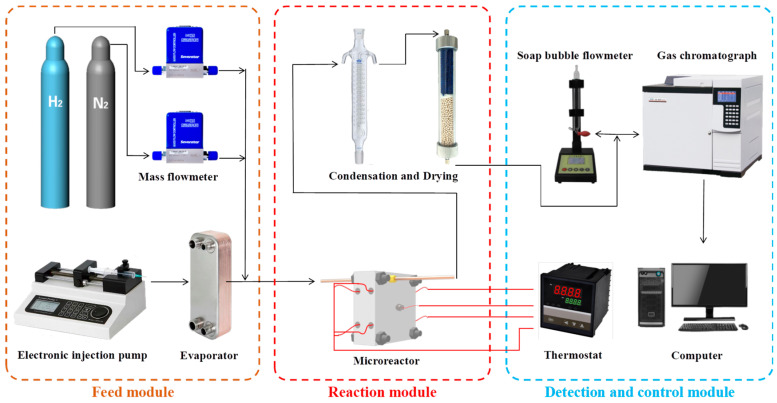
Testing system for the hydrogen production performance of the methanol steam reforming microreactor.

The hydrogen production performance test system of the methanol steam reforming microreactor loaded with the ceramic microchannel catalyst support is shown in Figure 3. Firstly, N_2_, as a protective gas with a flow rate of 50 mL/min, was injected into the microreactor, and the temperature of the microreactor was raised to 250 °C. H_2_ with a flow rate of 50 mL/min is then injected to activate the catalyst for 1 h. Then, the methanol–water mixture with a water–alcohol ratio of 1.3:1 was input into the test system. The methanol water mixture was vaporized and then fed into a microreactor for a reforming reaction in order to produce hydrogen-rich gas. The chemical equation for hydrogen production through the use of methanol steam reforming is as follows:

(1) $$SR:\;CH_{3}OH+H_{2}O\to 3H_{2}+CO_{2}\;∆H_{298}^{0}=49.4\;kJ/mol$$

(2) $$DE:\;CH_{3}OH\to 2H_{2}+CO\;∆H_{298}^{0}=92.0\;kJ/mol$$

(3) $$WGS:\;CO+H_{2}O↔H_{2}+CO_{2}\;∆H_{298}^{0}=-41.1\;kJ/mol$$

The hydrogen-rich gas was condensed and dried, and was then sent to the gas chromatograph (No: GC1690) for analysis. The hydrogen-rich flow rate was measured with a soap film flowmeter. The methanol conversion rate *MC* and hydrogen production rate *HPR* can be calculated as follows:

(4) $$MC=\frac{m_{n,out}(\varphi_{co}+\varphi_{{co}_{2}})}{m_{nMeOH,in}}\times 100\%$$

(5) $$HPR=m_{n,out}\times \varphi_{H_{2}}$$

where *m*_n,out_ is outlet molar flow; *φ* is the gas–volume ratio; and *m*_nMeOH,in_ is the inlet molar flow rate of methanol.

## 3. Results and Discussion

### 3.1. Microchannel Structure and Its Surface Morphology

**Figure 4:**
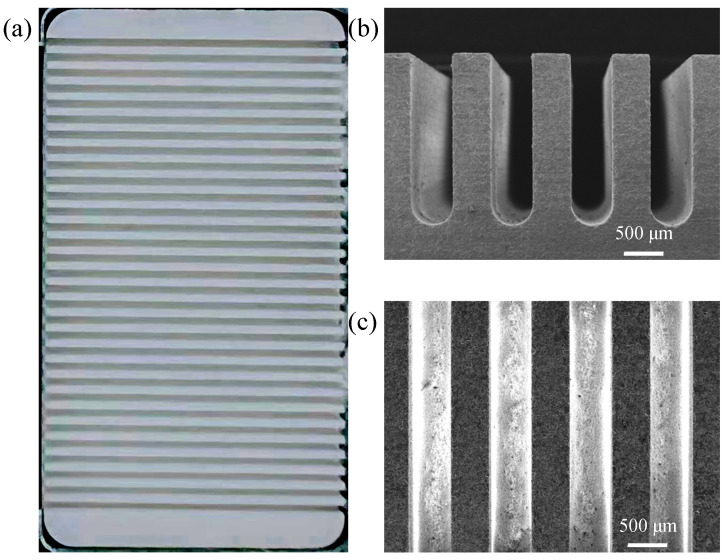
(**a**) Ceramic microchannel catalyst support processed through diamond wire sawing; (**b**) SEM image of microchannel cross-section; (**c**) Top view SEM image of microchannels.

Figure 4a shows the ceramic microchannel catalyst support that has been processed through diamond wire sawing. The size of the ceramic support is 20 mm × 35 mm × 2 mm. A total of 31 microchannels were machined on the surface of the ceramic workpiece, and the width of the microchannels was about 500 μm, the depth was about 1500 μm, and the spacing of each microchannel was about 1000 μm. As seen in the cross-section and top view of the ceramic support in Figure 4b,c, the bottom of the microchannel processed via diamond wire sawing was circular, the structure of the microchannel was complete, no obvious edge breakage or damage was found, and no burrs were seen. The parallelism and consistency of the microchannels were very good. The experiment shows that it is feasible to use diamond wire sawing technology to process the microchannels on the surface of ceramic materials.

**Figure 5:**
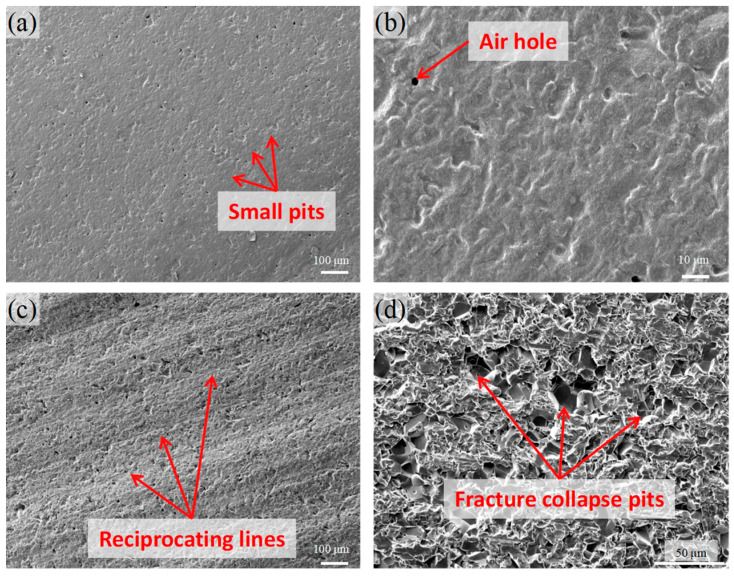
(**a**) SEM of sample 7-1 surface; (**b**) the details of sample 7-1 surface; (**c**) SEM of sample 4-4 surface; (**d**) the details of sample 4-4 surface.

There are two main ways of diamond wire sawing to remove materials, namely plastic removal and brittle removal. When the wire sawing speed is fast and the feed speed is relatively low, it is easy to remove plastic, and the material is scraped off by the abrasive particles in a plastic shear mode, thus forming a relatively smooth surface topography, as shown in Figure 5a. The surface of sample 7-1 was processed at a wire speed of 9.52 m/s and a feed speed of 6 μm/s. It can be seen that there were no obvious broken marks on the surface of the sample and that the surface was relatively smooth, with only some small pits and pores, which might be left over from the sintering process of ceramic materials.

When the wire speed is slow and the feed speed is fast, the cutting depth of a single abrasive particle will increase, and the cutting force generated by the abrasive particle will increase. Because the fracture toughness of hard and brittle materials like ceramic is generally low, the large cutting force can often cause the brittle fracture of certain materials, and then the surface morphology of the brittle collapse is formed. As shown in Figure 5c, Samples 4-4 were processed at a wire speed of 5.71 m/s and a feed speed of 24 μm/s. Due to the large cutting force, on the one hand, the reciprocating grain formed by the reciprocating motion of the saw wire can be seen on the surface, and, on the other hand, it can be observed that the surface of the ceramic material has obvious brittle collapse morphology. Among them, the formation of the reciprocating grain is related to the direction change of the force during the positive and negative rotation of the wire wheel. Because there is a certain dislocation in the winding process of the diamond saw wire, which leads to the saw wire, in addition to the normal direction of the cutting force applied to the material, moving in the direction perpendicular to the normal phase, there is also a certain component force, so that the path of the saw wire in the cutting process is not completely a straight line, but will periodically swing near the theoretical trajectory, forming a similar corrugated surface topography, that is, a reciprocating grain. In the general slicing process, a reciprocating grain is to be avoided as much as possible because it increases the roughness of the slice surface and may reduce the yield of the wafer. However, in the processing of the ceramic microchannel support, the reciprocating corrugated microstructure can increase the surface roughness and is more conducive to the adhesion of the catalyst. Similarly, the brittle collapse morphology accompanying the formation of the reciprocating grain will also significantly increase the surface area of the support, which may increase the solidity of the catalyst adhesion.

### 3.2. Effect of Processing Parameter on Periodic Corrugated Microstructure

**Figure 6:**
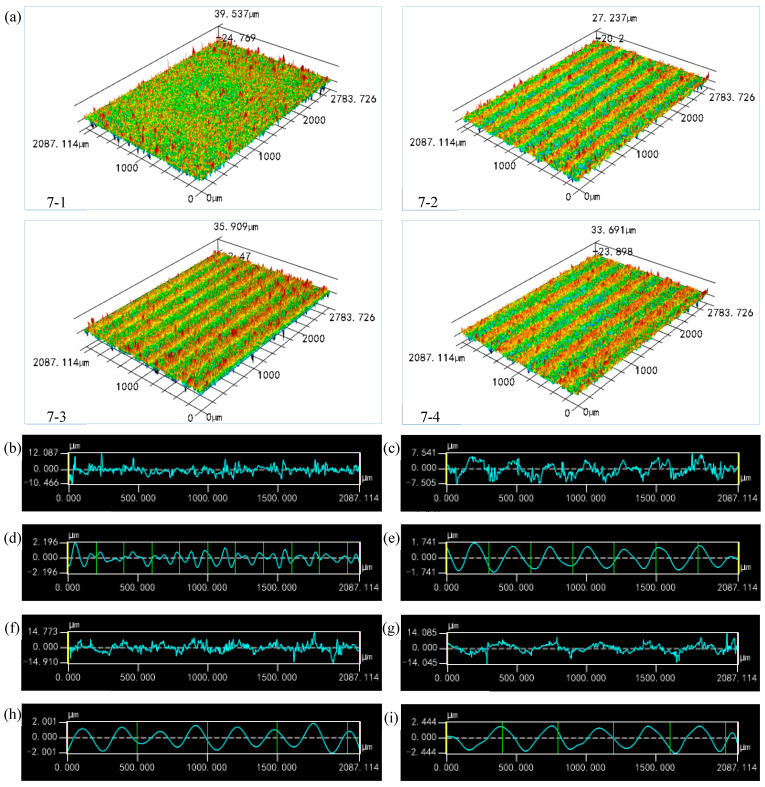
(**a**) Surface morphology of the ceramic microchannel catalyst support processed via diamond wire sawing under different feed speeds; (**b**) Surface profile of sample 7-1; (**c**) Surface profile of sample 7-2; (**d**) Waviness of sample 7-1; (**e**) Waviness of sample 7-2; (**f**) Surface profile of sample 7-3; (**g**) Surface profile of sample 7-4; (**h**) Waviness of sample 7-3; (**i**) Waviness of sample 7-4.

Figure 6a shows the 3D laser confocal scanning topography of the ceramic workpiece surface, processed via changing the feed speed under the condition of a constant wire speed. As can be seen from the figure, as the feed speed increased, the cyclic wave-like reciprocating grain became more obvious, and the wavelength and amplitude of the corrugated microstructure increased. It is worth noting that, when the feed speed was level 1, no obvious periodic corrugated structure was observed when compared with other feed speeds. This may be because, on the one hand, when the feed speed was very low, the cutting force decreased, and the removal mode of the material changed from brittle collapse to plastic shear, meaning it was not easy to form reciprocating ridges on the surface. On the other hand, with the decrease in the feed speed, the wavelength and amplitude of the periodic corrugated structure were reduced, and the waviness might coincide with the range of roughness; the waviness did not show obvious periodicity due to the interference of the surface roughness. Figure 6b–i are the surface topography profiles of the ceramic workpieces and their corresponding waviness at different feed speeds. It can be seen that, except for sample 7-1, the waviness of other samples shows a strong periodicity.

**Figure 7:**
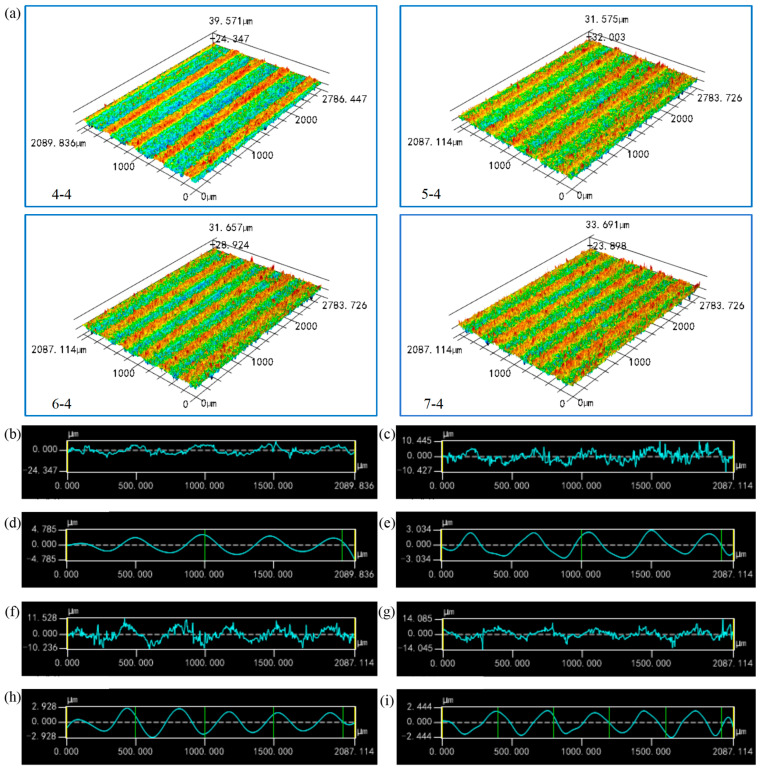
(**a**) Surface morphology of the ceramic microchannel catalyst support processed via diamond wire sawing under different wire speeds; (**b**) Surface profile of sample 4-4; (**c**) Surface profile of sample 5-4; (**d**) Waviness of sample 4-4; (**e**) Waviness of sample 5-4; (**f**) Surface profile of sample 6-4; (**g**) Surface profile of sample 7-4; (**h**) Waviness of sample 6-4; (**i**) Waviness of sample 7-4.

Figure 7a shows the 3D laser confocal scanning topography of the ceramic workpiece surface processed via changing the wire speed under the condition of a constant feed speed. Contrary to the rule of feed speed, increasing the sawing speed can obtain a periodic corrugated structure with smaller wavelength and amplitude. This is because, according to the cutting force analysis, the size of the cutting force is positively correlated with the reciprocal of the sawing speed. Therefore, increasing the sawing speed is conducive to reducing the cutting force, as well as also reducing the force component of the sawing wire deviating from the theoretical motion path, and the wavelength and amplitude of the formed corrugated microstructure are smaller. Figure 7b–i are the surface topography profiles of the ceramic workpieces and their corresponding waviness at different wire speeds. Due to the selection of the feed speed at level 4, the value was large, and all of the sample surface was observed to form an obvious periodic corrugated structure.

**Figure 8:**
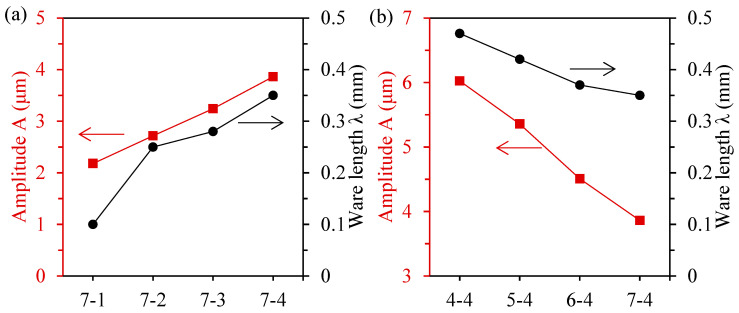
(**a**) Amplitude and ware length of the ceramic support surface processed via diamond wire sawing under different feed speeds; (**b**) Amplitude and ware length of the ceramic support surface processed via diamond wire sawing under different wire speeds.

In Figure 8, the average amplitude and wavelength of the surface waviness of each sample were extracted to analyze the influence of the processing parameters on the characteristic size of the corrugated microstructure. Due to the smooth surface of sample 7-1, the extracted data might not be representative, so they were used as reference only. It can be seen from Figure 8a that both the amplitude and wavelength of the microstructure increased with the increase in the feed speed. The amplitude increased from 2.71 μm for sample 7-2 to 3.86 μm for sample 7-3 by about 42.4%. The wavelength of sample 7-2 increased from 0.25 mm to 0.35 mm of sample 7-3 by about 40%. Figure 8b shows the variation trend of the amplitude and wavelength of the microstructure at different wire speeds. With the increase in the wire speed, the amplitude of the microstructure decreased significantly from 6.02 μm to 3.86 μm, which decreased by 35.9%. The ware length of the microstructure decreased from 0.47 mm to 0.35 mm, which decreased by 25.5%.

### 3.3. Effect of Periodic Corrugated Microstructure on Catalyst Coating Performance

**Figure 9:**
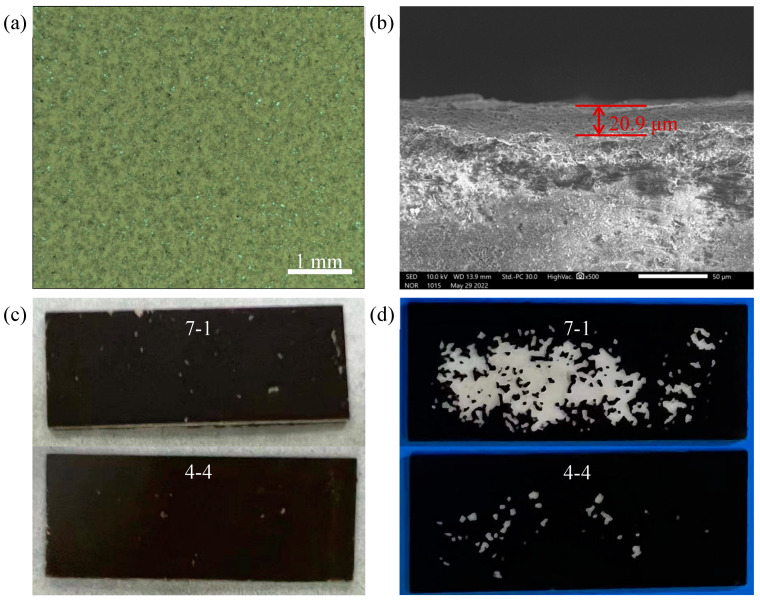
(**a**) Optical microscope observation of the catalyst coating surface; (**b**) SEM of the ceramic slice section after loading catalyst; Catalyst coating on the ceramic surface (**c**) before and (**d**) after air blowing.

In order to explore the influence of the characteristic parameters of the corrugated microstructure on the catalyst loading performance, better processing parameters should be sought for the processing of the ceramic catalyst support. The catalyst solution was loaded onto the surface of the processed ceramic sample with a multiple impregnation method after drying and calcining, as shown in Figure 9a. It can be seen that the catalyst could be more uniformly attached to the surface of the ceramic workpiece using the method of multiple impregnation. As can be seen from the SEM diagram in Figure 9b, the catalyst load thickness was about 20.9 μm, the coating thickness was relatively uniform, and no obvious separation phenomenon was found between the coating and the ceramic workpiece. In the actual working conditions, the catalyst needs to be continuously washed by the reaction gas, so the reliability of its load is crucial. The shedding and stripping of catalysts during the reaction may weaken the catalytic performance of the coating, and may even block the microchannel, thus affecting the efficiency of the hydrogen production reaction. Therefore, the strong wind purge experiment was used to test the adhesion properties of catalysts with different corrugated microstructures. It can be seen from Figure 9c,d that the surface of sample 7-1 was relatively smooth. After being swept by the strong wind for 90 s, the catalyst coating on the surface appeared to fall off in a large area. However, in sample 4-4 with a periodic corrugated microstructure on the surface, after being swept by the strong wind for 90s, the catalyst dropped only sporadically, and most of the catalyst was still firmly attached to the surface of the ceramic support.

**Figure 10:**
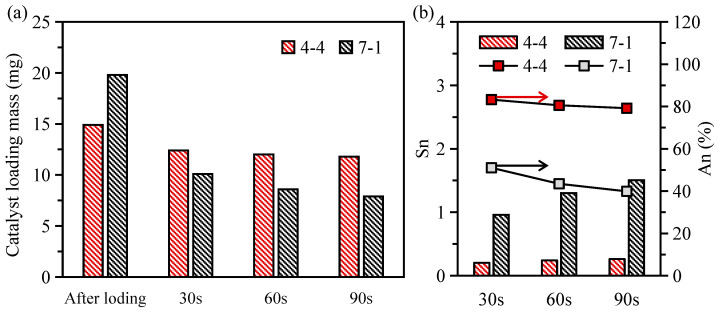
(**a**) Influence of the strong wind blowing time on the loading mass; (**b**) Influence of the strong wind blowing time on the catalyst shedding ratio and adhesion ratio.

Figure 10a shows the loading capacity of the catalyst after loading and after the strong wind blowing for 30 s, 60 s and 90 s. It can be seen that the initial catalyst load of sample 7-1 was larger than that of sample 4-4. This may be because the surface of sample 7-1 was relatively smooth, the surface tension was small, and the surface of the catalyst solution carrier was not easy to spread. After agglomeration and drying, the loading capacity of sample 7-1 was relatively high. The catalyst loading mass of both samples decreased with the increase in blowing time. However, the reduction in the catalyst loading mass in sample 7-1 appeared to be more severe than that in sample 4-4. In order to be able to quantify the analysis of the catalyst load performance, herein, the shedding factor *S*_n_ was defined as the ratio of the shedding catalyst mass to the remaining catalyst mass. A smaller shedding factor means less catalyst shedding. The expression is as follows:

(6) $$S_{n}=\frac{m_{n-1}-m_{n}}{m_{n}}$$

where *m* is the catalyst mass on the sample and *n* is the number of times of strong wind blowing.

The adhesion ratio *A*_n_ was defined as the ratio of the remaining catalyst mass after the *n*th blowing and the catalyst initial mass. The higher the adhesion ratio, the stronger the ability of the catalyst adhesion on the surface of the support. The expression is as follows:

(7) $$A_{n}=\frac{m_{n}}{m_{0}}\times 100\%$$

It can be seen from Figure 10b that, after 90 s of blowing, the shedding factor of sample 4-4 was maintained at about 0.25, and the adhesion ratio was as high as 80%. This shows that the surface of the support with the periodic corrugated structure demonstrated a good catalyst adhesion ability, which making it less likely to fall off. This can be attributed to the fact that, on the one hand, the periodic corrugated structure increased the specific surface area of the support, which increased the payload area of the catalyst. On the other hand, the periodic corrugated structure also formed a similar mechanical interlock structure between the ceramic substrate and the catalytic layer, which enhanced the adhesion ability of the catalyst. In contrast, the shedding factor of sample 7-1 with a smooth surface reached almost 1.5 after 90 s of blowing. And the adhesion ratio of it was only about 40%, which meant that nearly half the mass of the initial catalyst was lost. This may be because, on the one hand, when sample 7-1 was initially loaded, it was difficult to spread the catalyst solution due to the surface hydrophobicity, and the catalyst load was abnormally high. Although some catalysts seemed to be loaded onto the surface of the support, the contact between each other was not firm, meaning it was more likely to fall off. On the other hand, due to the smooth surface of the sample, the adhesion between the catalyst layer and the ceramic material substrate was realized through a weak van der Waals force, so it showed a bad catalyst loading performance during the strong wind blowing.

**Figure 11:**
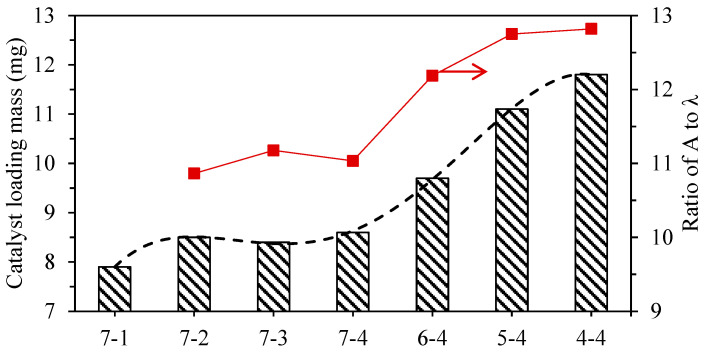
Relationship of the catalyst loading mass and the ratio of A to λ.

The relationship between the catalyst load performance and the characteristic parameters of the corrugated structure is shown in Figure 11. The weather wavelength or amplitude cannot fully reflect the characteristics of the corrugated microstructure. Here, the ratio of the amplitude and wavelength was defined as a parameter index to reflect the fluctuation degree of the corrugated structure. The smaller the value of A/λ, the more gentle the peaks and troughs of the corrugated structure, which may make it more similar to a plane, which is obviously not conducive to the adhesion of the catalyst. The greater the value of A/λ, the more violent the fluctuation of the corrugated structure, which is then more likely to form a mechanical interlock structure with the catalyst coating, thereby enhancing the load strength of the catalyst. From this point of view, we calculated the catalyst loads of different samples and their A/λ. The results show that the coating performance of the catalyst was consistent with the variation trend of its A/λ. Under the processing parameters in this experiment, it seems that changing the wire speed during processing can significantly affect the catalyst load of the sample.

### 3.4. Performance Evaluation of Methanol Steaming Reforming

**Figure 12:**
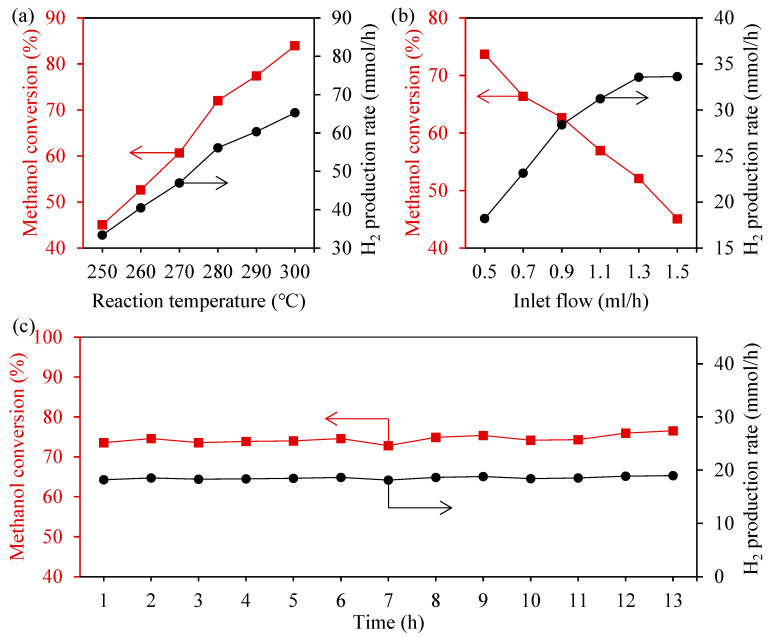
The methanol conversion rate and hydrogen production rate of the microchannel reactor: (**a**) Different reaction temperatures under an inlet flow of 1.5 mL/h; (**b**) Different inlet flows under 250 °C; (**c**) Stability test lasting 13 h under an inlet flow of 0.5 mL/h and 250 °C.

The processing parameters of sample 4-4 were used to process a catalyst support with 31 microchannels to test its hydrogen production performance via methanol steam reforming. The finished support will be loaded with approximately 0.15 g of the catalyst, then loaded into the microreactor, and finally into the test system for testing. In this test, the hydrogen production process was observed through controlling the reaction temperature and the flow rate of the inlet methanol solution. Figure 12a shows the variation trend of the methanol conversion rate and hydrogen production flow rate of the microreactor at different reaction temperatures when the inlet flow rate is 1.5 mL/h. It can be seen that methanol has a strong endothermic reaction. With the increase in temperature, the methanol conversion rate gradually increased and reached a maximum value of 84.0% at 300 °C, showing a good conversion efficiency. As the catalyst used for the hydrogen production of methanol reforming is a copper catalyst, its high temperature thermal stability is poor. When the temperature is higher than 300 °C, the catalyst is prone to agglomeration, carbon deposition, and other phenomena, resulting in catalyst failure. Therefore, the maximum temperature of this experiment is only 300 °C. Similarly, with the increase in methanol conversion, hydrogen production also increased and reached a maximum at 300 °C, which could produce about 65.3 mmol of hydrogen per hour.

Figure 12b shows the variation trend of the methanol conversion rate and hydrogen production flow rate of the microreactor under different inlet flow rates at the reaction temperature of 250 °C. It can be seen that, with the increase in the inlet flow, the conversion rate of methanol gradually decreased. This may be because, with the increase in the inlet flow rate, the flow rate of reactants increases, and the residence time in the microreactor decreases, resulting in partial methanol water vapor flowing out of the reaction area before it has time to fully react. In contrast, the hydrogen production increased as the inlet flow rate increased, and then the increment flattened out. This may be because the increase in the inlet flow increases the probability of collision between the reactant molecules and catalyst molecules, resulting in an increase in total hydrogen production. However, the surface area of the catalyst is not infinite but limited, as there is a catalytic limit; when the molecular collision reaches the limit, continuing to increase the amount of reactants can not improve the collision probability, so the hydrogen production gradually no longer increases significantly after reaching a certain degree.

Figure 12c shows the stability experiment when the reaction temperature is 250 °C and the inlet flow rate is 0.5 mL/h. It can be seen that, during the 13 h stability experiment, the conversion rate of methanol was basically maintained at about 75% without a significant reduction, and the hydrogen production was also maintained at 20 mmol/h. This shows that, during this period, the catalyst basically did not fall off, still being very firmly attached to the surface of the microchannel, and played a catalytic role. The experiments proved the effectiveness and stability of the corrugated microstructure for the enhancement of the catalyst load strength.

## 4. Conclusions

In this paper, a ceramic microchannel catalyst support with a periodic corrugated microstructure was fabricated through diamond wire sawing technology, and the catalyst loading performance of the support was enhanced by adjusting the characteristic parameters of the periodic corrugated microstructure, which was applied to the hydrogen production microreactor of methanol steam reforming, achieving good effect. The specific conclusions were as follows:

1. The feasibility of the machining ceramic microchannel catalyst support with diamond wire sawing was verified. On a ceramic plate with a size of 20 mm × 35 mm × 2 mm, 31 microchannels with a width of about 500 μm, a depth of about 1500 μm, and a spacing of about 1000 μm were fabricated. The structure of the processed microchannel was complete, and there was no obvious edge breakage or damage on the front and side. The parallelism and consistency of the microchannels were good.
2. The surface of the ceramic samples processed with different processing parameters was observed and analyzed using a 3D confocal laser microscope. The results show that the periodic corrugated microstructure with a larger amplitude and wavelength can be obtained through decreasing the wire speed and increasing the feed speed. In this experiment, when the wire speed and the feed speed are both level 4, the periodic corrugated microstructure with maximum amplitude and wavelength can be obtained, with an amplitude of 6.02 μm and a wavelength of 0.47 mm.
3. The ceramic support was loaded with the catalyst via multiple impregnation. The loading strength of the catalyst was tested using strong wind blowing. The experimental results show that the ceramic support with a periodic corrugated microstructure displays better catalyst loading performance. The sample still has 80% of the adhesion ratio after strong wind blowing lasting 90 s. A/λ is defined as a characteristic parameter evaluation index of the periodic corrugated microstructure. The coating performance of the catalyst was consistent with the variation trend of its A/λ.
4. A hydrogen production performance test showed that ceramic support with a periodic corrugated microstructure presents good hydrogen production performance. When the inlet flow rate was 1.5 mL/h and the reaction temperature was 300 °C, the methanol conversion rate was as high as 84%, and the hydrogen production rate was 65.3 mmol/h. When the inlet flow rate is 0.5 mL/h and the reaction temperature is 250 °C, after a 13 h stability test, the hydrogen support performance remains basically unchanged.

The experimental results of this paper provide a new idea and theoretical guidance for the manufacturing technology of a ceramic catalyst support and its application in the field of hydrogen energy. Optimizing the processing parameters (including feed speed, wire speed, wire diameter, reciprocating period, etc.) to obtain the optimal periodic corrugated microstructure morphology which can maximize the efficiency of catalyst loading and hydrogen production will be an important direction of future research.

## Figures and Tables

**Table 1:** Comparison of various processing methods.

Methods	Processing Efficiency	Advantages	Disadvantages
Injection molding	Medium	Simple process; High precision; Low cost	Mold manufacturing expansive; Demolding difficulty
Laser machining	Low	Path programmable	Expensive; Low precision
EDM	Very low	Complex shape processing; High precision	Very time consuming
Micro-milling	Low	High precision; Path programmable	Size limit; Time consuming
DWS	High	Simple process; High precision; Low cost	Path non-programmable

**Table 2:** The material parameters of Al_2_O_3_ ceramic.

Density (g/cm^3^)	Hardness	E (GPa)	Poisson’s Ratio	Fracture Toughness (MPa·m^1/2^)
3.85	HRA 85	463	0.22	4

**Table 3:** Processing parameters.

Levels	Wire Speed (mm/s)	Levels	Feed Speed (μm/s)
1	95	1	6
2	286	2	12
3	476	3	18
4	571	4	24
5	667	5	30
6	762		
7	952		

**Table 4:** Experiment parameters.

Parameters	Wire Speed (mm/s)	Feed Speed (μm/s)
Group 1	952 (Lv.7)	6, 12, 18, 24 (Lv.1 2 3 4)
Group 2	571, 667, 762, 952 (Lv.4 5 6 7)	24 (Lv.4)

## Data Availability

The raw data supporting the conclusions of this article will be made available by the authors on request.
